# Phenomics-Assisted Selection for Herbage Accumulation in Alfalfa (*Medicago sativa* L.)

**DOI:** 10.3389/fpls.2021.756768

**Published:** 2021-12-07

**Authors:** Anju Biswas, Mario Henrique Murad Leite Andrade, Janam P. Acharya, Cleber Lopes de Souza, Yolanda Lopez, Giselle de Assis, Shubham Shirbhate, Aditya Singh, Patricio Munoz, Esteban F. Rios

**Affiliations:** ^1^Department of Agronomy, University of Florida, Gainesville, FL, United States; ^2^EMBRAPA-ACRE, Rio Branco, Brazil; ^3^Department of Agricultural and Biological Engineering, University of Florida, Gainesville, FL, United States; ^4^Department of Horticultural Sciences, University of Florida, Gainesville, FL, United States

**Keywords:** high-throughput phenotyping (HTP), normalized difference vegetation index (NDVI), remote sensing (RS), spatial variation, genetic gain, forage, plant breeding

## Abstract

The application of remote sensing in plant breeding is becoming a routine method for fast and non-destructive high-throughput phenotyping (HTP) using unmanned aerial vehicles (UAVs) equipped with sensors. Alfalfa (*Medicago sativa* L.) is a perennial forage legume grown in more than 30 million hectares worldwide. Breeding alfalfa for herbage accumulation (HA) requires frequent and multiple phenotyping efforts, which is laborious and costly. The objective of this study was to assess the efficiency of UAV-based imagery and spatial analysis in the selection of alfalfa for HA. The alfalfa breeding population was composed of 145 full-sib and 34 half-sib families, and the experimental design was a row-column with augmented representation of controls. The experiment was established in November 2017, and HA was harvested four times between August 2018 and January 2019. A UAV equipped with a multispectral camera was used for HTP before each harvest. Four vegetation indices (VIs) were calculated from the UAV-based images: NDVI, NDRE, GNDVI, and GRVI. All VIs showed a high correlation with HA, and VIs predicted HA with moderate accuracy. HA and NDVI were used for further analyses to calculate the genetic parameters using linear mixed models. The spatial analysis had a significant effect in both dimensions (rows and columns) for HA and NDVI, resulting in improvements in the estimation of genetic parameters. Univariate models for NDVI and HA, and bivariate models, were fit to predict family performance for scenarios with various levels of HA data (simulated *in silico* by assigning missing values to full dataset). The bivariate models provided higher correlation among predicted values, higher coincidence for selection, and higher genetic gain even for scenarios with only 30% of HA data. Hence, HTP is a reliable and efficient method to aid alfalfa phenotyping to improve HA. Additionally, the use of spatial analysis can also improve the accuracy of selection in breeding trials.

## Introduction

Alfalfa (*Medicago sativa* L.) is the most important perennial forage legume globally because of its relatively high yield and nutritional value (Annicchiarico, 2015). In the United States, alfalfa is the fourth most valued crop behind corn, soybeans, and wheat, with an estimated value of $8.4 billion (USDA-NASS, 2020), playing a critical role in the food supply chain (Feng et al., 2020). In 2018, nearly 53 million tons of alfalfa and alfalfa-grass mixtures were harvested from almost seven million hectares in the United States. Most of the production is concentrated in the mid-east and west coast (USDA-NASS, 2020). Despite its lower presence as a forage crop in the lower southeastern United States and other subtropical regions in the world, breeding efforts are underway to develop non-dormant alfalfa cultivars adapted to these environments (De Assis et al., 2010; Vivela et al., 2018; Adhikari et al., 2019; Acharya et al., 2020).

Alfalfa breeding is typically conducted as phenotypic recurrent selection using among and within half-sib family selection (Casler and Brummer, 2008), although various breeding schemes have been proposed to improve herbage accumulation (HA) in alfalfa (Annicchiarico and Pecetti, 2021). The improvement of HA in alfalfa is challenging due to long selection cycles, tetrasomic inheritance, high inbreeding depression, and significant genotype and environment interaction for this complex trait (Bingham et al., 1994; Brummer, 1999; Annicchiarico, 2015). Additionally, phenotyping for HA requires investment of significant resources (Annicchiarico et al., 2016). In recent years, most alfalfa breeding programs have focused on improving disease/pest resistance, long-term persistence, and other specific traits targeting transgenes for glyphosate tolerance or decreased lignin. The lack of efforts to improve HA can explain the low genetic gain in alfalfa yield observed in the last decades (Brummer and Casler, 2015).

Nevertheless, HA has become a target breeding trait among alfalfa breeders more recently (Sakiroglu and Brummer, 2017; Dos Santos et al., 2018; Adhikari et al., 2019; Acharya et al., 2020; Benabderrahim et al., 2020; He et al., 2020; Ren et al., 2021; Tang et al., 2021). However, traditional field phenotyping for HA is based on the destructive sampling of experimental units at the ground level, weighing fresh samples, drying, and weighing dried samples to estimate dry matter content. The manual phenotyping process for HA is labor-intensive, time-consuming, and costly.

Plant phenotyping plays a central role in plant breeding, and the accurate and rapid acquisition of phenotypic data is valuable for exploring the association between genotypes and phenotypes. In the last few decades, remote sensing has been widely used in agriculture (Maes and Steppe, 2019; Galli et al., 2020), particularly for high-throughput phenotyping (HTP) in breeding applications (Furbank and Tester, 2011; White et al., 2012; Araus and Cairns, 2014; Tattaris et al., 2016; Li et al., 2017; Zhao et al., 2019). Remote sensing offers unprecedented spectral, spatial, and temporal resolution, providing detailed vegetation data (Maes and Steppe, 2019). Several vegetation indices (VIs) such as normalized difference vegetation index (NDVI), green NDVI (GNDVI), normalized difference red edge (NDRE), or Green and Red ratio Vegetation Index (GRVI) have been employed to assess vegetation vigor and canopy cover over multiple crops (Lima-Cueto et al., 2019; Quirós Vargas et al., 2019; Ranjan et al., 2019; Zhang et al., 2019).

Remote sensing techniques have shown to enable efficient and non-destructive estimation of HA in alfalfa (Feng et al., 2020), such as screening large breeding populations (Cazenave et al., 2019). According to Cazenave et al. (2019), HTP can detect small differences in alfalfa yield when screening diverse germplasm. More recently, HTP improved the efficiency of the selection process for biomass in small plots (1.52 m × 0.30 m) in alfalfa breeding populations (Tang et al., 2021), and provided a good prediction of HA in larger plots (6 m × 4 m) (Feng et al., 2020). Remote sensing can mitigate the challenge of measuring HA in large populations for breeding programs focusing on improving alfalfa HA. Therefore, the implementation of HTP can streamline the phenotyping process for HA in alfalfa.

Residual maximum likelihood (REML) is commonly implemented in breeding programs to estimate variance components and calculate genetic parameters using linear mixed models. The use of best linear unbiased prediction (BLUP) is an established technique to predict breeding values, which are then used to guide breeding decisions. BLUP can generate accurate predictions of breeding values even for unbalanced experimental designs (Piepho et al., 2008). Genetic parameters for alfalfa yield are essential to define optimal selection schemes (Casler and Brummer, 2008). Heritability estimates for HA in alfalfa ranged from 0.15 to 0.30 (Bowley and Christie, 1981; Riday and Brummer, 2002; Annicchiarico, 2015; Acharya et al., 2020). These low to moderate estimates are expected in alfalfa and other perennial forage crops with long selection cycles, cross-pollinated breeding schemes, and traits with significant genotype × environment interaction (Annicchiarico, 2015). Most studies in alfalfa have focused on yield in short-term experiments with few harvests (3–4 harvests per year) (Annicchiarico, 2015), except for more recent studies (Acharya et al., 2020; Annicchiarico and Pecetti, 2021). Besides the challenges mentioned above, field trials are associated with intrinsic and extrinsic variations, which can cause some form of spatial variation between experimental units (Sripathi et al., 2017). Local control, such as blocking and randomization, cannot effectively account for all the spatial trends in large experiments (Gilmour et al., 1997). Spatial variation is expected even using complex experimental designs, such as those commonly used in most plant breeding programs. A better way to control the spatial variation is to implement spatial analysis to detect and correct the variation patterns in multiple dimensions. Experimental units close to each other are expected to be higher correlated than those far apart, and improvements in model fitness and higher selection accuracy have been reported in plant breeding programs (Andrade et al., 2020).

The overall objective of our research was to implement HTP, spatial analysis, and linear mixed models to improve the accuracy of the selection process in alfalfa for HA. The specific objectives were: (i) phenotype of an alfalfa breeding population for HA using ground-based manual sampling and utilize a unmanned aerial vehicle (UAV) for HTP; (ii) assess the efficiency of controlling field variation using spatial models for HA and NDVI in alfalfa, (iii) calculate the genetic parameters based on HA and NDVI using univariate models; (iv) fit bivariate models for HA and NDVI using all data, and for scenarios with different levels (30–90%) of HA data, and (v) quantify the correlation between breeding values, the coincidence of selection of the best families, and genetic gain across the different scenarios for HA data.

## Materials and Methods

### Germplasm Screening and Development of the Reference Breeding Population

Initially, 121 alfalfa populations with different fall dormancy groups were screened for HA in Citra, FL, United States (Acharya et al., 2020). A total of 33 populations were selected based on high HA and persistence across all harvests. Following the screening, controlled crosses were done in the greenhouse to create the alfalfa reference breeding population. A single plant per population was selected based on vigor, and cuttings were made in the summer 2016. A factorial mating design was used to create all possible full-sib combinations; however, some crosses did not produce enough viable seed, and were not included in field trials. Half-sib seed were also harvested from each parental line. All crosses were conducted in controlled conditions in the Forage Breeding and Genetics Lab greenhouse, at University of Florida (Gainesville, FL, United States). Seeds from each full-sib and half-sib families were harvested, threshed individually, planted in 72-cell Styrofoam trays in August 2017, and maintained in the greenhouse until transplanting in November 2017. In total, 145 full-sib and 33 half-sib families were established in the field and this population represents the reference set for the HTP study. Acharya et al. (2020) provide more details and results for the initial screening and crosses.

### Experimental Design and Field Management

The breeding trial with the reference population was conducted at Citra, Florida (29°40′ N, 82°167′ W, 48 m above the sea level) following a row and column design with augmented representation of controls. Each experimental unit (1.82 m × 1.82 m) consisted of eight rows spaced at 22.8 cm. The three border rows on each side were seeded with the Bulldog 805 to serve as borders, and twenty alfalfa seedlings were transplanted in the middle two rows. Three rows were seeded with Bulldog 805 on each side of the transplants to serve as borders. The breeding population was composed of 145 full-sib and 34 half-sib families. Three controls were used: the cultivars Bulldog 805, Florida 99, and an advanced breeding line named UF_AP_2015. Eighty-one families were replicated three times, 61 families were replicated two times, and 40 families were used one time due to limited seed availability. The experiment was established in November 2017, and data collection occurred in August 2018, October 2018, December 2018, and January 2019. The field was fertilized with 67.25 kg⋅K_2_O⋅ha^–1^, using Muriate of Potash, and with Boron at the rate of 1.12 kg ha^–1^ and herbicide Clethodim (Select, 70.76 g AI/L-1; Valent United States Corporation, Walnut Creek, CA, United States) was applied to control grasses at the rate of 1.05 kg ha^–1^ after each harvest. Manual weeding was done as needed to control broad-leaf weeds after each harvest.

### Ground-Based Data Collection

**FIGURE 1:**
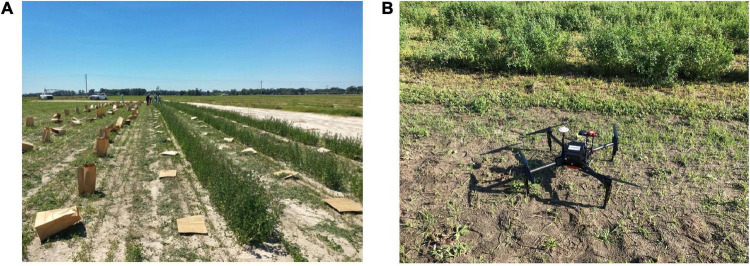
Phenotyping alfalfa for: **(A)** ground-based herbage accumulation (HA); and **(B)** High-throughput phenotyping (HTP) using an unmanned aerial vehicle (Matrice 100) with multispectral camera (MicaSense RedEdge, Seattle, WA, United States) taking off in the experimental area.

The experimental units were manually harvested to determine HA (kg ha^–1^) when the control UF_AP_2015 reached 10% blooming (Figure 1A). The harvest was performed by mowing the six outer rows (three rows on each side) with a flail mower at 10 cm stubble height, and then the two central rows were cut and weighted to determine the fresh weight (g) by the plot. Approximately 500 g of fresh shoots were collected from each plot and placed in a dryer at 55°C for 7 days to determine dry matter content, and HA per plot was estimated on a dry matter basis (kg ha^–1^).

### Remote Sensing Data Collection

A UAV (DJI Matrice 100) equipped with a multispectral camera (RedEdge, MicaSense, Seattle, WA, United States) was used to obtain imagery over the entire field after the border rows were mowed (Figure 1B). AtlasFlight app (MicaSense Inc., Seattle, WA, United States) was used to automatically sample fields at an altitude of 30 m, a flight speed of 6 m/s speed, and enforcing a 75% overlap in collected imagery. A calibration panel (MicaSense Inc., Seattle, WA, United States) was set before starting each flight to allow post-collection calibration of imagery.

### Image Processing and Data Acquisition

**FIGURE 2:**
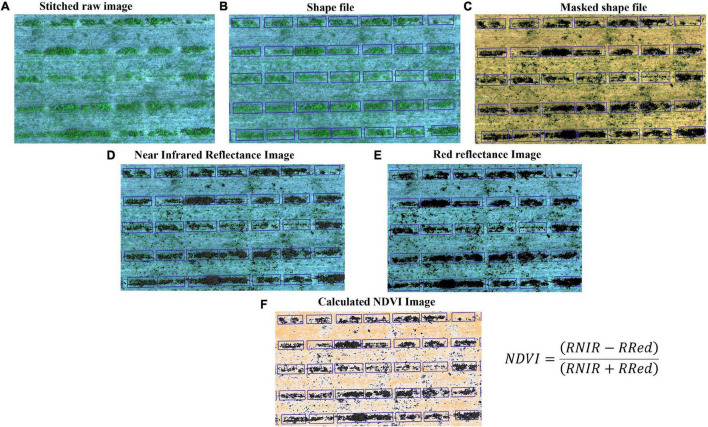
Workflow of image processing and data acquisition for the HTP. **(A)** Stitched raw images (each row is visible) from multispectral camera, **(B)** Shape file: each plot is separated by grid line, **(C)** Masked shapefile: canopy and bare ground places are noticeable, and these images are used to generate vegetation indices, **(D)** Reflectance image of infrared band, **(E)** Reflectance image of red band, and **(F)** Calculated NDVI image using panels **(D,E)**.

All images were stitched into orthomosaics using AgiSoft Photoscan (Agisoft LLC, St. Petersburg, Russia; Figure 2A). The orthomosaic corresponded to the entire field for a single harvest event and comprised five bands: blue (475 nm), green (560 nm), red (668 nm), near-infrared (NIR, 840 nm), and red edge (717 nm). The orthomosaics were further processed in QGIS 3.14 software (QGIS.org 2020) to refine geolocation using field-collected ground control points. We identified experimental units from imagery and created a spatial reference frame (ESRI Shapefile) for further analysis (Figure 2B). The shapefile was edited to include all relevant information on the individual field plot. Subsequently, we masked the canopy from the soil using custom python codes (Figure 2C) and used the masked image to calculate the total pixel count in each band (Figures 2D–F) and to generate different Vis (NDVI, GNDVI, NDRE, and GRVI) from zonal statistics function in QGIS. We also estimated the sum of all VIs to allow for handling plants that died during the experiment.

### Data Analysis

#### Vegetation Index Selection

We utilized boxplots to assess all VIs and HA distributions across each harvest (Supplementary Figure 1). We estimated Pearson correlations between HA and VIs-sum (an integrative indicator of VIs) in R (R Core Team, 2020) to assess prospective best-fitting relationships between HA and VIs. Finally, we used ordinary linear mixed models to model HA using VIs-sum for each harvest (R Core Team, 2020).

#### Variance Component Estimation: Base Model

Linear mixed models were fit using the package ASReml-R (Butler et al., 2009) in the software R (R Core Team, 2020). The significance of random effects was determined by the likelihood ratio test. Univariate models were fit for NDVI and HA by harvest, as follows:


(1) $$\text{y}=\mu +\text{Xt}+{\text{Z}}_{r}\,{\text{u}}_{r}+{\text{Z}}_{c}\,{\text{u}}_{c}+{\text{Z}}_{f}\,{\text{u}}_{f}+\text{e}$$

where ***y*** is the vector of the response variable, μ is the overall mean; t is the fixed effect vector of the check varieties; **u_r_** is the random effect vector of the row, **u_r_**∼ N (0, **I** $\sigma_{r}^{2}$); **u_c_** is the random effect vector of the column, **u_c_**∼ N (0, **I** $\sigma_{c}^{2}$); **u_f_** is the random effect vector of the family, assuming that families are independent **u_f_**∼ N (0, **I** $\sigma_{f}^{2}$), and **e** is the independent error random vector of residual, *e* ∼ N (0, **I** $\sigma_{e}^{2}$). I is the identity matrix associated with the vector, while X, **Z_r_**, **Z_c_**, and **Z_f_** represent the incidence matrices associated with the vectors *t*, **u_r_**, **u_r_**, and **u_f_**. The variance components of the effects ***r***, ***c***, ***f***, and ***e*** are represented by the $\sigma_{r}^{2}$, $\sigma_{c}^{2}$, $\sigma_{f}^{2}$, and $\sigma_{e}^{2}$, respectively.

#### Variance Component Estimation: Spatial Model

Due to the intrinsic variation in the field, we explored spatial models to account for spatial autocorrelation among experimental units. In this model, we assumed that the error term was auto correlated along the rows and columns, and we used a first-order autoregressive process to fit the error:


(2) $$\text{y}=\mu +\text{Xt}+{\text{Z}}_{r}\,\text{r}+{\text{Z}}_{c}\,\text{c}+{\text{Z}}_{f}\,\text{f}+\xi$$

where all terms are the same as the model (1) other than the term ξ, which is the independent error random vector of residual, ξ∼ N (0, R_*e*_$\sigma_{e}^{2}$), R_*e*_ is the covariance matrix of ξ, and it is defined as: R_*e*_ σξ^**2**^**Σ_c_**(ρ_**c**_)⊗**Σ_r_**(ρ_**r**_). Where ρ_**c**_ and ρ_**r**_ are the autocorrelation parameters for the spatial coordinates of row and column; **Σ_c_**(ρ_**c**_)and **Σ_r_**(ρ**_r_**) represent the autoregressive correlation matrices; and **⊗** represents the Kronecker product (Andrade et al., 2020).

From both base and spatial models, we estimated the following genetic and non-genetic parameters: broad-sense heritability (H^2^), predicted error variance (PEV), and relative efficiency (RE) between the spatial model and base model. The RE was calculated for the spatial model in relation to the base model based on PEV and values greater than 100 indicate higher efficiency for the spatial model. The RE was measured as follows:


(3) $$R\,E=100\times \left(\frac{P\,E\,V_{B\,a\,s\,e}}{P\,E\,V_{S\,p\,a\,t\,i\,a\,l}}\right)$$

The Akaike information criteria (AIC) for each model were used to choose the best model. Additionally, the families were ranked based on their predicted values from each model for the traits HA and NDVI.

#### Variance Component Estimation: Bivariate Model and Scenarios for Herbage Accumulation Data

Data for HA and NDVI were combined into a single model to leverage information at both levels (ground-based and HTP). As manual phenotyping for HA is time-consuming and costly, scenarios were simulated *in silico* to quantify how genetic parameter estimates would change when not all experimental units are manually harvested. The simulation was performed by randomly assigning missing values to the full HA dataset to represent hypothetical scenarios when 30, 40, 50, 60, 70, 80, and 90% of the plots would be harvested. The process was repeated 30 times for each scenario. The base model contained 100% of the HA data, and it was used as a baseline to compare with other scenarios. The bivariate model was fitted as follows:


(4) $$\text{y}=\mu +\text{Xt}+{\text{Z}}_{r}\,{\text{u}}_{r}+{\text{Z}}_{c}\,{\text{u}}_{c}+{\text{Z}}_{f}\,{\text{u}}_{f}+\text{e}$$

where **y** is a stacked vector of the phenotypic data for traits HA (t1) and NDVI (t2), μ is the stacked vector of the overall mean for each trait; t is the fixed effect stacked vector of the check varieties for each trait; **u_r_** is the random effect stacked vector of the row for each trait, **u_r_**∼ N (0, $\sigma_{r}^{2}$); **u_c_** is the random effect stacked vector of the column for each trait, **u_c_**∼ N (0, $\sigma_{c}^{2}$); **u_f_** is the random effect stacked vector of the family for each trait, assuming that families are independent **u_f_**∼ N (0, I_*f*_⊗**G**), G = $\left[\begin{array}{cc} \sigma_{ft1}^{2} & \sigma_{ft1ft2}\\ \sigma_{ft1ft2} & {}_{ft2}^{2} \end{array}\right]$; and *e* is the independent error random stacked vector of residual for each trait, **e**∼ N (0, I_*e*_⊗R), where R = $\left[\begin{array}{cc} \sigma_{e\,t\,1}^{2} & \sigma_{e\,t\,1\,e\,t\,2}^{2}\\ \sigma_{e\,t\,1\,e\,t\,2}^{2} & \sigma_{e\,t\,2}^{2} \end{array}\right]$; **X**, *Z_r_*, *Z_c_*, and *Z_f_* represent the incidence matrices associated with the vectors *t*, **u_r_**, **u_c_**, and **u_f_**. The components $\sigma_{r}^{2}$, $\sigma_{c}^{2}$, ${}_{f\,t\,1}^{2}$, ${}_{f\,t\,2}^{2}$, ${}_{e\,t\,1}^{2}$, and ${}_{e\,t\,2}^{2}$ are the variance components for row, columns, family for trait 1, family for trait 2, error for trait 1, and error for trait 2, respectively. The component_*ft1,t2*_ is the covariance between trait 1 and 2.

We compared the full bivariate and univariate models utilizing HA, NDVI with the different scenarios using the following calculations: (i) coincidence of selection (%) after applying a 10% selection intensity and (ii) the correlation among predicted values across all families.

Genetic gain (%) was estimated from BLUPs for each family in each harvest (Supplementary Table 3) for the bivariate model, and univariate models for HA and NDVI in all scenarios for HA missing data. We calculated genetic gain using the following equation:


(5) $$\text{Genetic }\,\text{Gain}=\frac{\bar{{\text{BLUP}}_{t}}}{\bar{\text{Y}}}\,\text{x }\,100$$

$\bar{B\,L\,U\,P_{t}}$is the mean of the BLUPs of the *t* selected family for HA, $\bar{Y}$ is the overall mean of all families for HA.

## Results

### Pearson Correlation and Regression Analysis Between Herbage Accumulation and Vegetation Indices

**FIGURE 3:**
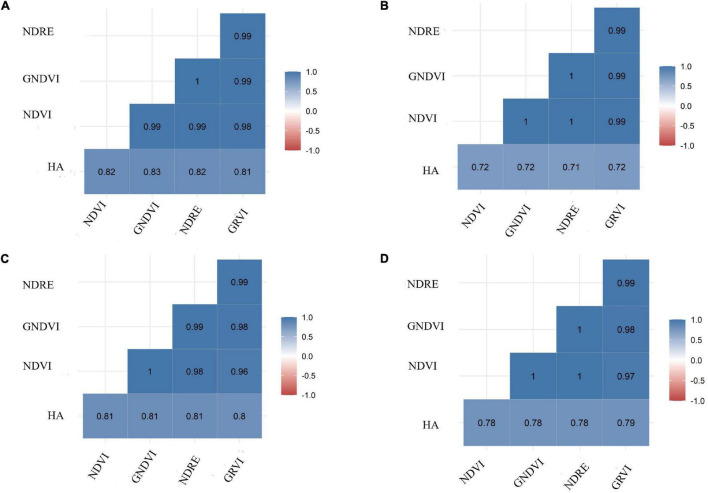
Pearson correlation coefficients between ground-based phenotyping for herbage accumulation (HA—kg ha^– 1^) and four vegetation indices (VIs): NDVI, normalized difference vegetation index; GNDVI, green normalized difference vegetation index; NDRE, normalized difference red edge; GRVI, green and red ratio vegetation index. Harvests: **(A)** harvest one, **(B)** harvest two, **(C)** harvest three, and **(D)** harvest four.

**FIGURE 4:**
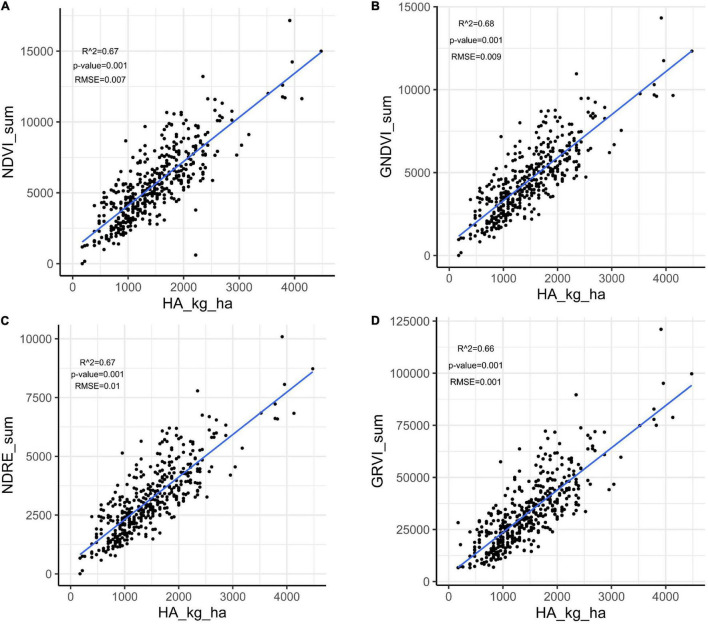
Linear regression between herbage accumulation (HA_kg_ha) and unmanned aerial vehicles-based VIs collected in harvest one in alfalfa families evaluated in Citra, FL. VIs: **(A)** NDVI, normalized difference vegetation index; **(B)** GNDVI, green normalized difference vegetation index; **(C)** NDRE, normalized difference red edge; GRVI, **(D)** green and red ratio VI.

Herbage accumulation showed variation across harvests (Supplementary Figure 1). Harvest one and four showed higher mean HA and variation, while harvest two had the lowest mean HA and variation. All VIs responded similarly to the variation in HA across harvests, as all VIs had a higher mean and variation for harvest one, and the lowest mean and variation in harvest two (Supplementary Figures 3A–E). The Pearson correlation between HA and all VIs (NDVI, GNDVI, NDRE, and GRVI) were higher than 0.71 across all four harvests (Figure 3). All VIs were able to model HA with moderate and similar accuracy across harvests (Figure 4 and Supplementary Figures 2–4). Harvest one, three, and four showed better prediction (*R*^2^ > 0.60) for HA than harvest two (R^2^ ∼ 0.51) (Figure 4 and Supplementary Figures 2–4). Due to the similar results observed among all VIs, NDVI was selected for further analyses.

### Spatial Analysis to Control Field Variation

**FIGURE 5:**
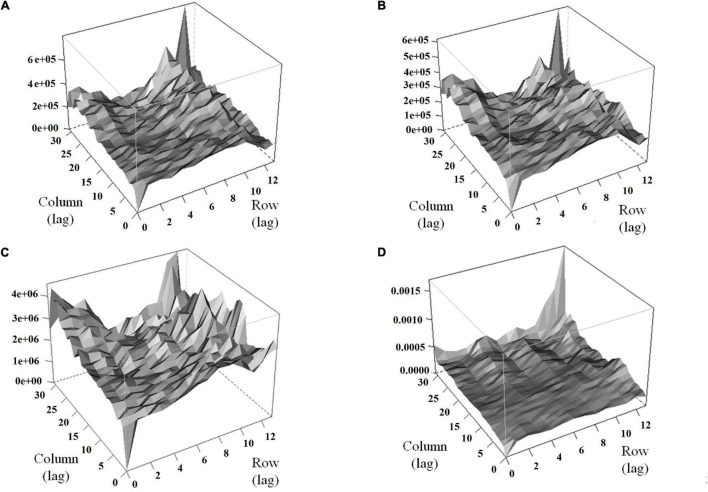
Variograms for HA and normalized difference vegetation index (NDVI) before **(A,C)** and after **(B,D)** the inclusion of terms to control local and global trends for alfalfa yield in harvest one in alfalfa families evaluated in Citra, FL. **(A)** HA-based model, **(B)** HA-spatial model, **(C)** NDVI-based model, **(D)** NDVI-spatial model. Row and column are coordinates for the rows and columns in the experimental area, respectively.

Modeling the spatial variation was necessary for both traits (HA and NDVI) since the autocorrelation in both dimensions was significant in all harvests (Supplementary Table 1). Variograms for the base model showed the presence of patterns in the field that may increase error variance (peaks in the variograms indicate trends in the field) (Figures 5A,C). The variograms revealed that the spatial model efficiently controlled these patterns (Figures 5B,D). The spatial models for HA and NDVI provided better model fitness across all harvests (lower AIC and BIC; Supplementary Table 2).

The genotypic variance was significant (*P* < 0.001) for HA and NDVI across harvests, and heritability (*H*^2^) estimates ranged from low (0.12) to moderate (0.31) (Table 1). For HA, the spatial model provided slightly higher *H*^2^ estimates than the base model across the harvests (Table 1). Similarly, NDVI models accounting for spatial variation resulted in higher *H*^2^ estimates, except for harvest four (Table 1). The *H*^2^ estimates for HA (base and spatial models) were higher than *H*^2^ estimates for NDVI (base and spatial models) across all harvests. As model fitness was greater for spatial models, PEVs were also smaller for spatial models for HA and NDVI across harvests, except for NDVI in harvest four (Table 1). The lower PEV in the spatial models yielded higher RE than the base model, for all traits and harvests, except for HA in harvest four. The best model for each trait and harvest was used for further analyses.

**TABLE 1:** Estimates of broad-sense heritability (*H*^2^), predicted error variance (PEV) and relative efficiency (RE) for alfalfa families harvested four times in Citra, FL, United States.

Harvest	Parameter	HA		NDVI	
		Base	Spatial	Base	Spatial
1	*H* ^2^	0.28***	0.31***	0.21***	0.29***
	PEV	31,145	29,976	413,474	368,663
	RE	–	103.9	–	112.2
2	*H* ^2^	0.18***	0.20***	0.14***	0.18***
	PEV	15,503	15,048	2,140	2,099
	RE	–	103.1	–	101.9
3	*H* ^2^	0.24***	0.27***	0.13***	0.19***
	PEV	19,785	19,442	54,468	51,576
	RE	–	101.8	–	105.6
4	*H* ^2^	0.19***	0.23***	0.12***	0.11***
	PEV	38,223	39,548	27,300	25,200
	RE	–	97.4	–	108.2

*Linear mixed models were fitted for herbage accumulation (HA) and normalized difference vegetation index to estimate variance components in a model without accounting for spatial variation (base) and by modeling the spatial variation (spatial) in each harvest.*

***denotes significance at p < 0.001 for the genetic variance using a Likelihood Ratio Tests (LRT).

### Selection of Best Alfalfa Families for Herbage Accumulation and Normalized Difference Vegetation Index

**FIGURE 6:**
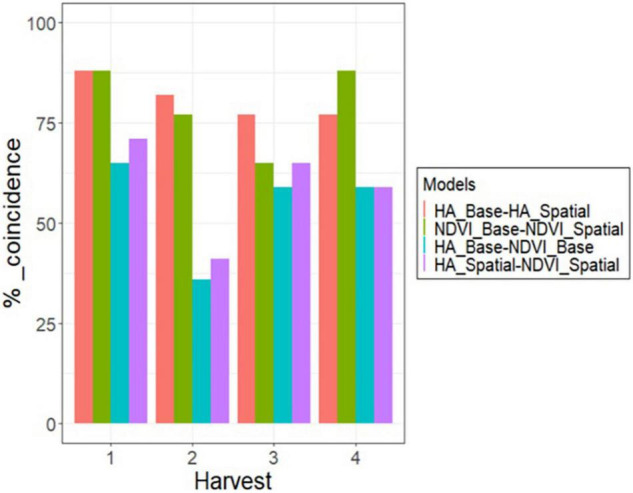
Percent of coincidence in selection for the 10% best alfalfa families between HA and NDVI, using the base and spatial model, across four harvests in alfalfa families evaluated in Citra, FL, United States.

The 179 alfalfa families were ranked based on their predicted values estimated on each harvest for the base and spatial models. Then, a 10% selection intensity was imposed to select the best 17 families (highest HA and NDVI values in each harvest). The coincidence of selection was greater than 75% (13 families out of 17) for the base and spatial models for HA and NDVI in all harvests, except for NDVI in harvest three (Figure 6). The coincidence of selection between HA and NDVI, based on the base model, ranged from 36% in harvest 2–65% in harvest one (Figure 6). The coincidence of selection between spatial models for HA and NDVI ranged from 41% in harvest 2–71% in harvest one (Figure 6).

Harvest one showed the highest coincidence of selection for all model comparisons (Figure 6). Base and spatial models for HA and NDVI resulted in 89% coincidence (15 families out of 17) (Figure 6). Considering the base model in harvest one, selecting families using HA and NDVI showed 65% coincidence (11 families). After modeling spatial variation, there was a 71% coincidence (12 families) when the best families were selected based on HA and NDVI. Harvest two showed the lowest coincidence when comparing the selected families for HA and NDVI using base and spatial models (Figure 6). Considering the base model in harvest two, selecting families using HA and NDVI showed 35% coincidence (five families). There was a 43% coincidence (seven families) for the spatial models to select the best 10% families based on HA and NDVI data. Harvests three and four showed similar results to harvest one, but slightly lower coincidence when comparing HA and NDVI for base and spatial models.

### Univariate and Bivariate Models for Scenarios With Different Levels of Herbage Accumulation Data

**FIGURE 7:**
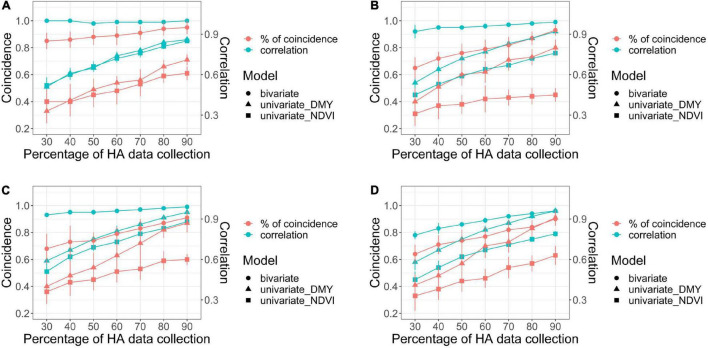
Comparison of bivariate and univariate models (shape) for HA and NDVI for coincidence of selection after applying a 10% selection intensity (red), and correlation among breeding values for all families (green), for scenarios comparing models with increasing levels of HA data collection (30–90%) against a model with 100% HA data, across four harvests in alfalfa families evaluated in Citra, FL, United States. Harvests one **(A)**, two **(B)**, three **(C)**, and four **(D)**.

**FIGURE 8:**
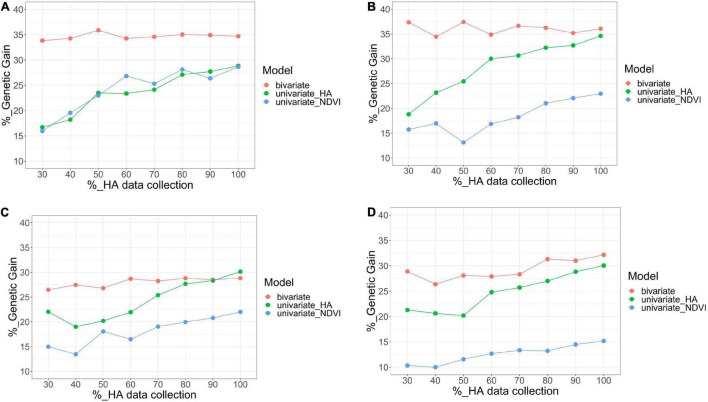
Comparison of bivariate model (HA and NDVI, red), and univariate (HA, green; NDVI, blue) models for genetic gain for HA, after applying a 10% selection intensity for scenarios with increasing levels of HA data collection (30–100%) across four harvests in alfalfa families evaluated in Citra, FL, United States. Harvests one **(A)**, two **(B)**, three **(C)**, and four **(D)**.

The combination of HA and NDVI data into a bivariate analysis was compared to the univariate models for each trait, considering scenarios with various levels of HA data for three parameters: correlation among predicted values across all families, the coincidence of selection for the 10% best families, and genetic gain. In general, all parameters increased as the level of HA data increased in all harvests (Figures 7, 8).

For the bivariate model, the correlation between the predicted values for all families using the complete HA dataset and each scenario with various levels of missing HA data (30–90%) varied between 0.78 (harvest four at 30% HA data collection) and 1 (harvest one at 90% HA data collection) (Figure 7). The correlation was consistently higher than 0.90 for three harvests (one, two, and three), even for the scenario when only 30% of HA data were used in the model. The coincidence of selection for the best 10% families varied between 0.64 (harvest four at 30% HA data collection) and 0.85 (harvest one at 90% HA data collection) (Figure 7). The correlation and coincidence of selection were consistently higher for the bivariate model than any univariate model across all scenarios (Figure 7). The bivariate and univariate models for HA were similar only in harvests three and four, for scenarios when 80 and 90% of the HA data were used in the model (Figure 7). The genetic gain for the bivariate model was higher than the univariate models for HA and NDVI in all harvests (except in harvest three for HA in the scenarios when 80 and 90% of the HA data were used in the model), and it remained stable even for scenarios with low levels of HA data (Figure 8).

For the univariate model for HA, the correlation between the genotypic values among all families varied between 0.51 (harvest one at 30% HA data collection) and 0.91 (harvest four for 90% HA data collection) (Figure 7). The coincidence of selection varied between 0.37 (harvest three at 30% HA data collection) and 0.84 (harvest two at 90% HA data collection) (Figure 7). The genetic gain for the univariate model for HA increased as more HA data was used in the models across all harvests, and higher gains were obtained for HA compared to NDVI for almost all scenarios (Figure 8). For the univariate model for NDVI, the correlation between genotypic values among all families varied between 0.45 (harvests two and four at 30% HA data collection) and 0.88 (harvest three for 90% HA data collection) (Figure 7). The coincidence for selection varied between 0.33 (harvest four at 30% HA data collection) and 0.63 (harvest four at 90% HA data collection) (Figure 7). In general, the univariate model for HA provided higher correlations and % coincidence than univariate models for NDVI (Figure 7), and lower genetic gain for HA was obtained when the selection was performed using only NDVI data (Figure 8).

## Discussion

The ultimate goal in plant breeding is to select superior breeding units (individuals, clones, families, etc.) with the highest accuracy level in a high throughput manner by investing the least possible resources. Alfalfa breeders aim to develop superior cultivars with high yield and quality, exhibiting broad adaptation to various biotic and abiotic stresses. Breeding programs are focusing on the improving HA invest significant resources in collecting and quantifying HA from field trials and drying samples to determine their dry matter content (Annicchiarico, 2015). This process is time-consuming and expensive for large breeding populations. A key component for increasing the efficiency in improving HA yield is the use of fast and precise phenotypic assessment of large breeding populations (Fu, 2015). In this study, 179 alfalfa families were phenotyped for HA across four harvests, totaling 1,792 data points for HA. At the same time, HTP was implemented to assess the efficiency of HTP to predict alfalfa HA. All VIs provided a high correlation with HA, and HA in alfalfa was modeled with moderate accuracy (*R*^2^ > 0.66 in four harvests). These results follow similar trends from the previous studies evaluating the efficiency of HTP in predicting HA in small plots from alfalfa germplasm and breeding lines (Cazenave et al., 2019; Tang et al., 2021), as well as larger alfalfa plots (Feng et al., 2020).

The progress in plant breeding is measured based on genetic gain, which refers to the amount of increase in performance achieved through cycles of artificial selection (Xu et al., 2017). Several factors affect genetic gain, such as the genetic variation available in breeding populations, trait heritability, selection intensity, and the time required to complete a breeding cycle (Xu et al., 2017). Estimation of heritability can be improved by refining field experiments and statistical approaches, particularly for understanding and controlling spatial variation. One of our goals was to evaluate the effect of spatial models to control field variation and improve the estimation of genetic parameters and family selection. The autocorrelation had a significant impact across rows and columns. The spatial models improved the estimation of genetic and non-genetic parameters for HA in all harvests and NDVI in harvests one, two, and three. After applying spatial analysis, the heritability increased for both HA and NDVI. Similarly, Sripathi et al. (2017) and Andrade et al. (2020) reported high efficiency of spatial analysis in the estimation of genetic parameters in potato and forage breeding populations. These authors reported improvements in model fitness from the base and to spatial, which supports our results. The results presented in our study showed the importance of the spatial model to reduce the PEV and improve selection accuracy. These results reflected higher precision in the selection of the best families. The spatial models for HA and NDVI showed high levels of coincidence of selection in all harvests (>75%, except for NDVI in harvest three), compared to the base model.

Plant breeders can increase the selection intensity through improvements in the scale and precision of genotyping and phenotyping, which will result in higher genetic gain (Xu et al., 2017). One of the strategies to improve selection intensity is by increasing the breeding populations’ size, but this comes at the expense of more efforts and resources dedicated to phenotyping. HTP can lead to higher genetic gain by increasing the size of breeding populations and making selections more accurately (Houle et al., 2010; Tang et al., 2021). In our study, *H*^2^ estimates were slightly lower for NDVI than HA, but both traits showed significant genetic variation and moderate to low *H*^2^. Considering only *H*^2^, NDVI was able to detect the genetic variation present in this breeding population and can be used to select breeding lines exhibiting higher NDVI values, which would translate to breeding lines with higher HA (*R*^2^ > 0.66). However, the coincidence of selection for the best families with HA and NDVI for both models was low to moderate (0.35 – 0.72), which shows that different families were selected by using NDVI and HA data in univariate models. Moreover, the genetic gain for HA was lower when the selection of the best 10% of the families was performed using only NDVI data. Our results indicated the NDVI data would complement ground-based HA measurements to improve genetic gain for HA in alfalfa.

Costs of field experiments are the limiting factor in alfalfa breeding programs focusing on quantifying HA across multiple harvests in a year and across multiple years and locations. The results presented in this study reported moderate to low *H*^2^ for HA and high correlation coefficients between HA and NDVI across harvests. Multi-trait selection can be applied to take advantage of the correlation between traits and increase selection accuracy for the target trait (Mrode, 2014). HA and NDVI data combined into a bivariate model for each harvest showed a higher correlation among predicted values, a higher coincidence of selection, and greater genetic gain than univariate models for HA and NDVI. As the level of HA data used in the models increased (from 30 to 90% of the total data), the correlation, coincidence of selection, and genetic gain increased. These results highlight the importance of collecting HTP data at the harvest time, particularly if breeders are not harvesting all experimental units in large breeding populations. To increase genetic gain for HA, alfalfa breeders could screen more breeding lines by combining HA and HTP phenotyping in their pipeline since the number of plots that need to be harvested will be smaller. In this study, reducing phenotyping efforts by 50% (using only 50% of the available HA data) showed a range between 0.83 and 1 for correlation among all families, 0.75 to 0.89 for the coincidence of selection, and 26.8 to 37.44% genetic gain in bivariate models. Despite the lower correlation and coincidence of selection, the genetic gain remained stable across all scenarios for bivariate models in all harvests. Including NDVI in the phenotyping pipeline for HA in alfalfa could result in greater genetic gains by increasing the size of breeding populations (Xu et al., 2017), while maintaining the resources for HA phenotyping constant.

High-throughput phenotyping is a promising method to develop improved cultivars and achieve high genetic gain. In this study, all VIs showed a high correlation with HA, and the inclusion of NDVI improved the selection accuracy for HA when bivariate models were fitted, even for scenarios with limited HA data. These results suggest that breeders could increase population size while maintaining the same ground-level measurement efforts, and expect increases in genetic gain due to a higher number of breeding candidates. Similar to the previous studies in alfalfa, HTP predicted HA with high accuracy (Feng et al., 2020), and HTP was able to detect differences in biomass production in large breeding populations (Cazenave et al., 2019). The results presented in this study coincide with the report from Tang et al. (2021), where HTP improved the efficiency of the selection process for alfalfa biomass in small plots (1.52 m × 0.30 m). Besides, it was also shown that spatial models controlled field variation and improved the estimation of genetic parameters and the accuracy of family selection.

Despite the improvements in the selection, HTP brings new challenges into the breeding pipeline. HTP data collection, storage, and processing require investments in computer power and storage and programming knowledge for data analysis and interpretation. In conclusion, the investment in time and resources to collect, process, and analyze HTP resulted in a more accurate selection of alfalfa families for HA. The RS data complemented ground-based HA measurements, and the combination of both datasets should result in improvements in alfalfa HA.

## Data Availability Statement

The data that support the findings of this study are available from the corresponding author upon request.

## Author Contributions

ER and PM designed the experiments. SS and AS collected the UAV images. JA, CS, YL, PM, and ER took the ground measurements. AB processed the images. AB and MA analyzed the data. GA and CS contributed to the data analysis on the ground measurements. AS, PM, and ER provided critical reviews and supported the costs of the study. AB, MA, and ER wrote the article. All authors provided critical reviews.

## Conflict of Interest

The authors declare that the research was conducted in the absence of any commercial or financial relationships that could be construed as a potential conflict of interest.

## Publisher’s Note

All claims expressed in this article are solely those of the authors and do not necessarily represent those of their affiliated organizations, or those of the publisher, the editors and the reviewers. Any product that may be evaluated in this article, or claim that may be made by its manufacturer, is not guaranteed or endorsed by the publisher.
