# Supplementary material for: Phenomics-Assisted Selection for Herbage Accumulation in Alfalfa (Medicago sativa L.)
Source: Front Plant Sci. 2021 Dec 7;12:756768. doi: 10.3389/fpls.2021.756768 (PMC8689394; doi:10.3389/fpls.2021.756768)
Supplement: Supplementary file 1 [file Data_Sheet_1.docx]

Supplementary Material

## Supplementary Figures


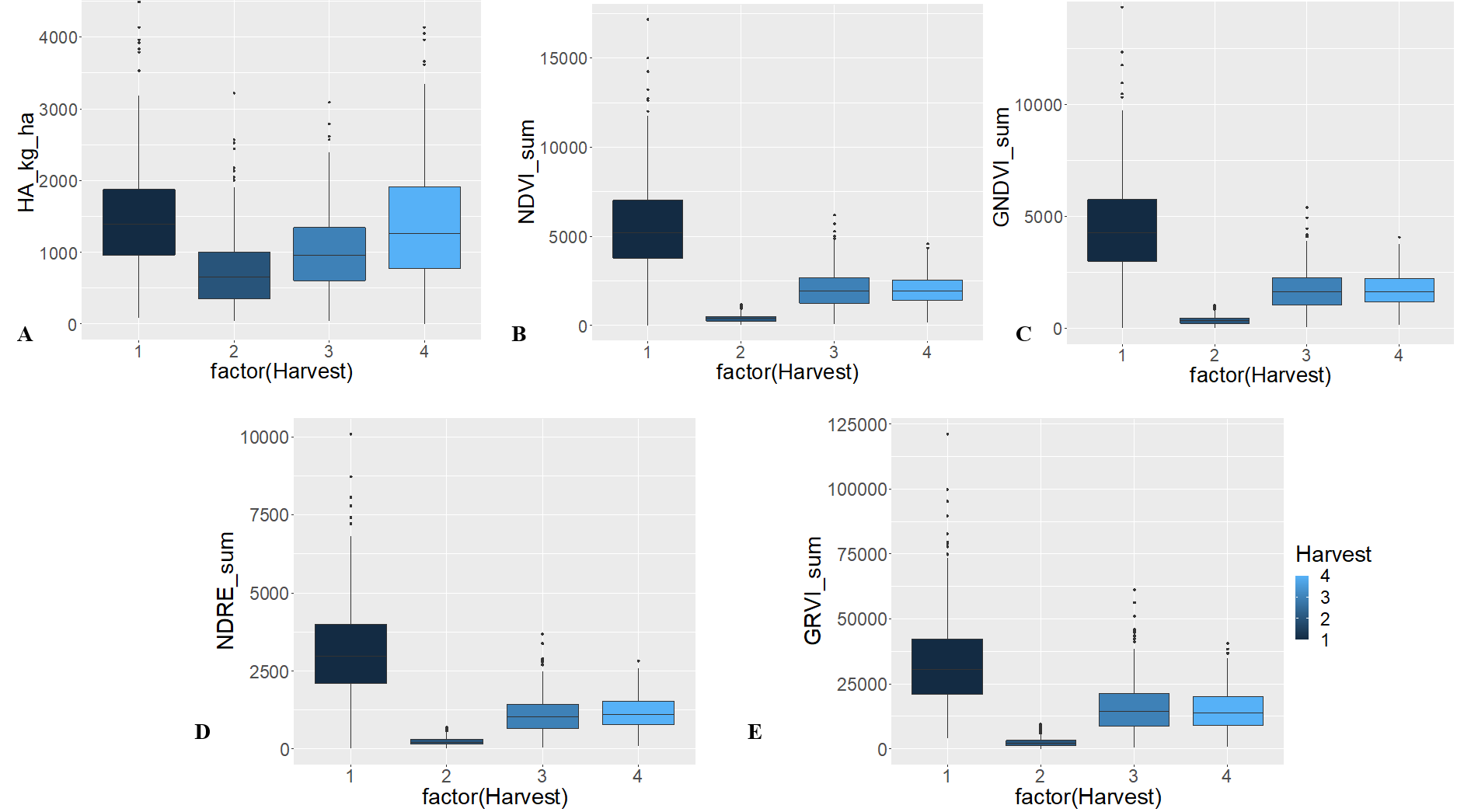


**Supplementary Figure 1.** Boxplots across four harvests in an alfalfa breeding trial conducted in Citra, FL. Traits: A) Ground-based: herbage accumulation (kg/ha), B) NDVI, normalized difference vegetation index, C) GNDVI, green normalized difference vegetation index, D) NDRE, normalized difference red edge, E) GRVI Green and Red ratio Vegetation Index.


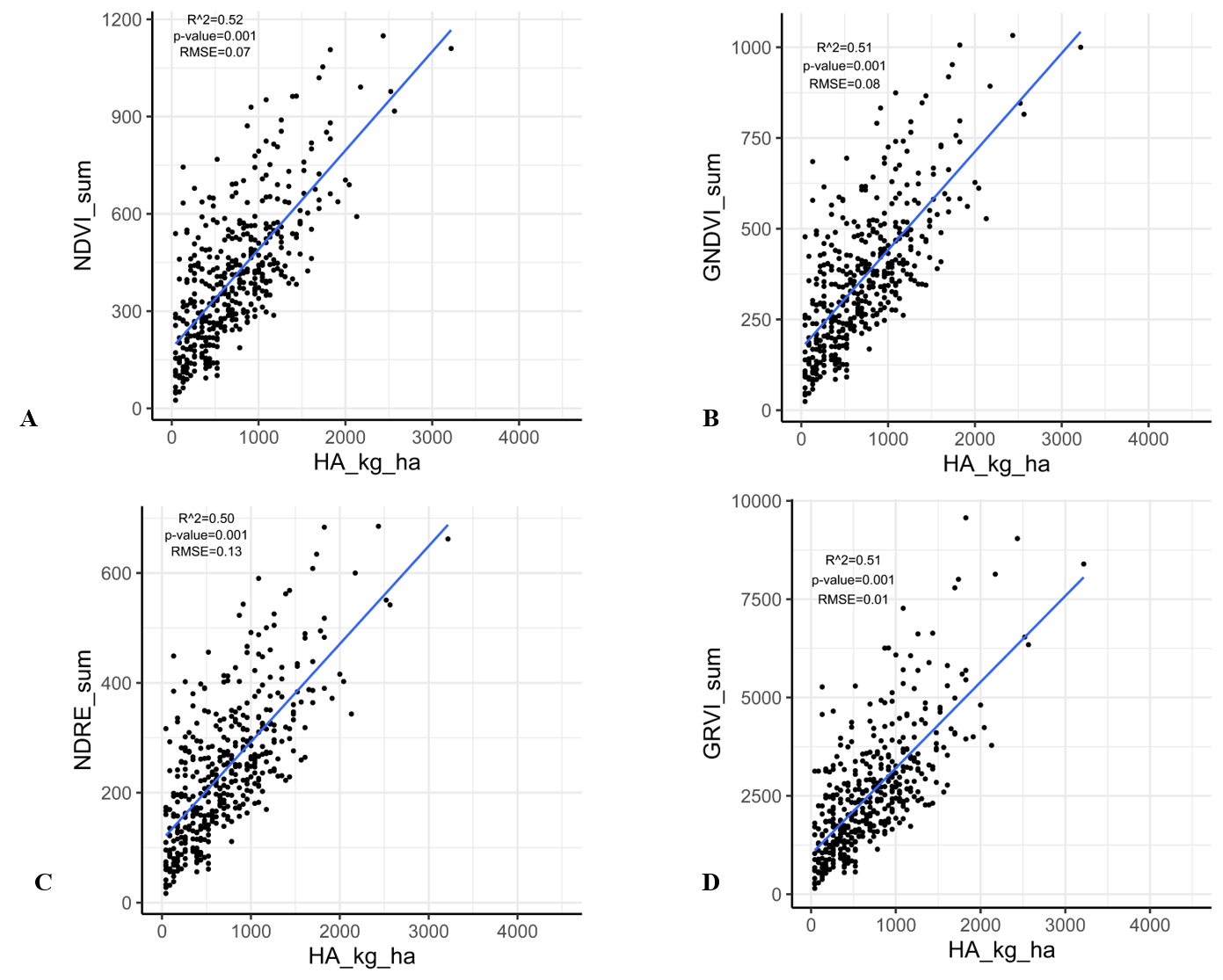


**Supplementary Figure 2.** Linear regression for herbage accumulation (HA) of alfalfa and UAV-based vegetation indices (VIs) collected in harvest number two from alfalfa breeding trial conducted in Citra, FL. VIs: (A) NDVI normalized difference vegetation index, (B) GNDVI, green normalized difference vegetation index, (C) NDRE, normalized difference red edge, GRVI, (D) Green and Red ratio Vegetation Index.


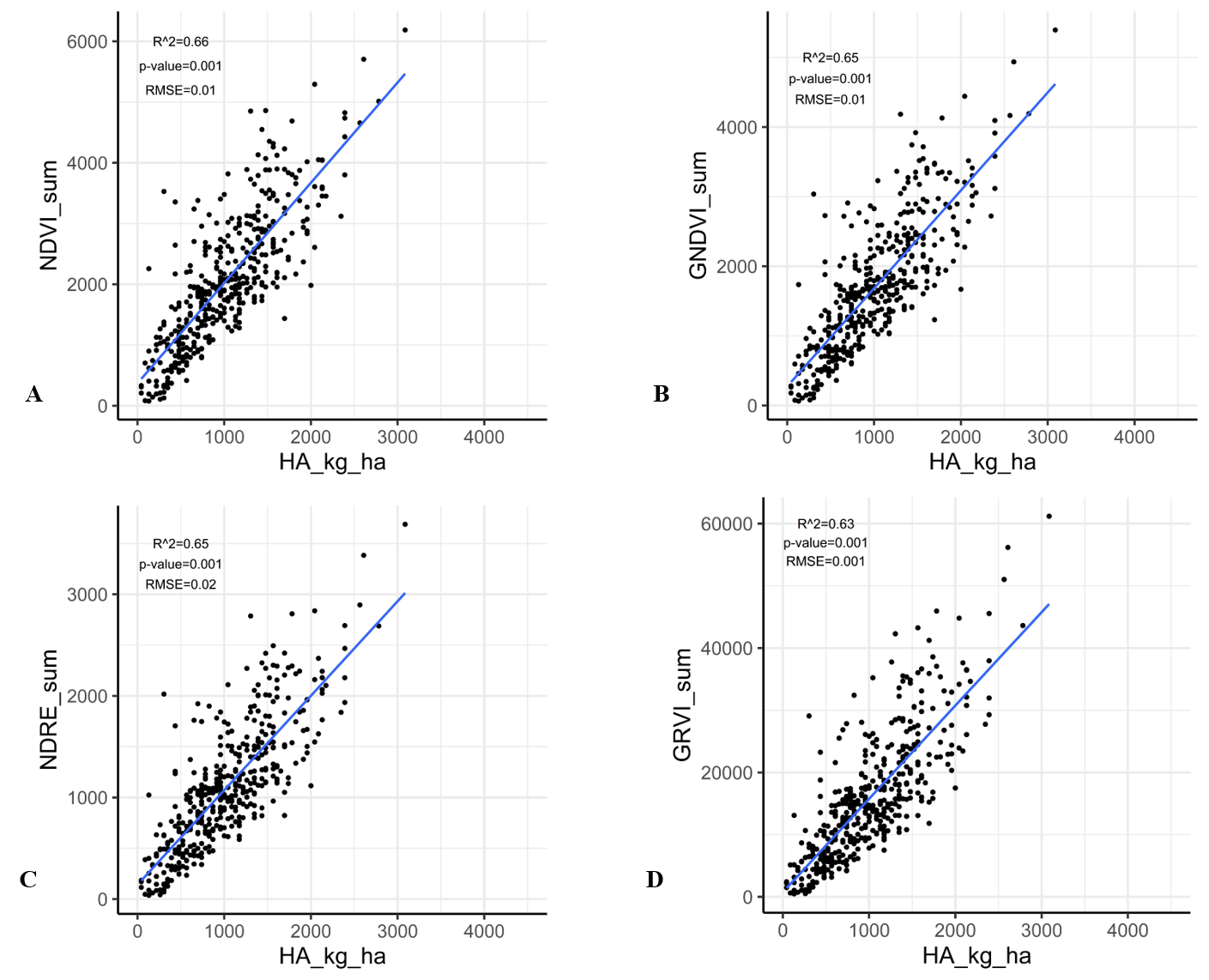


**Supplementary Figure 3.** Linear regression for herbage accumulation (HA) of alfalfa and UAV-based vegetation indices (VIs) collected in harvest number three from alfalfa breeding trial conducted in Citra, FL. VIs: (A) NDVI normalized difference vegetation index, (B) GNDVI, green normalized difference vegetation index, (C) NDRE, normalized difference red edge, GRVI, (D) Green and Red ratio Vegetation Index.


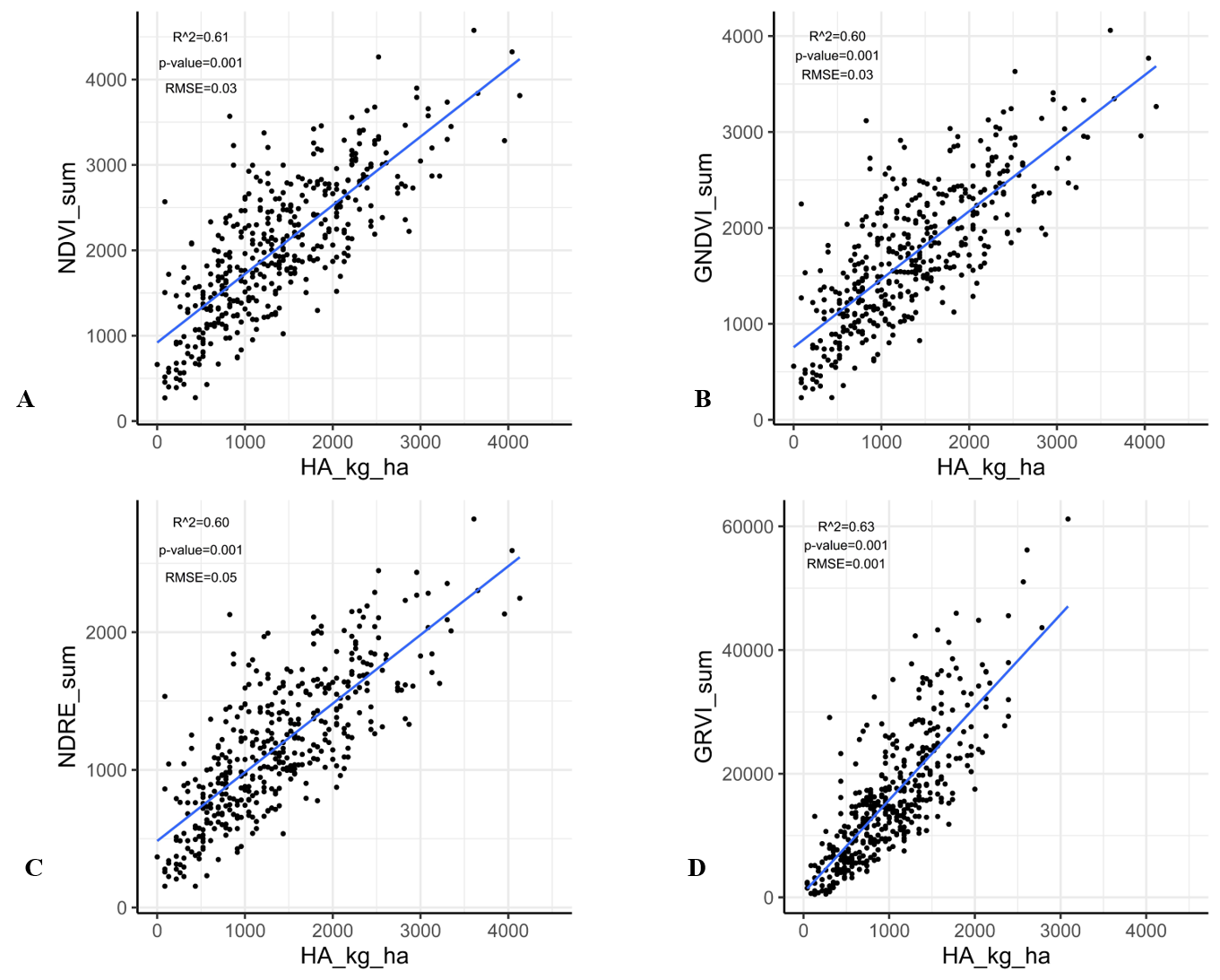


**Supplementary Figure 4.** Linear regression for herbage accumulation (HA) of alfalfa and UAV-based vegetation indices (VIs) collected in harvest number four from alfalfa breeding trial conducted in Citra, FL. VIs: (A) NDVI normalized difference vegetation index, (B) GNDVI, green normalized difference vegetation index, (C) NDRE, normalized difference red edge, GRVI, (D) Green and Red ratio Vegetation Index.


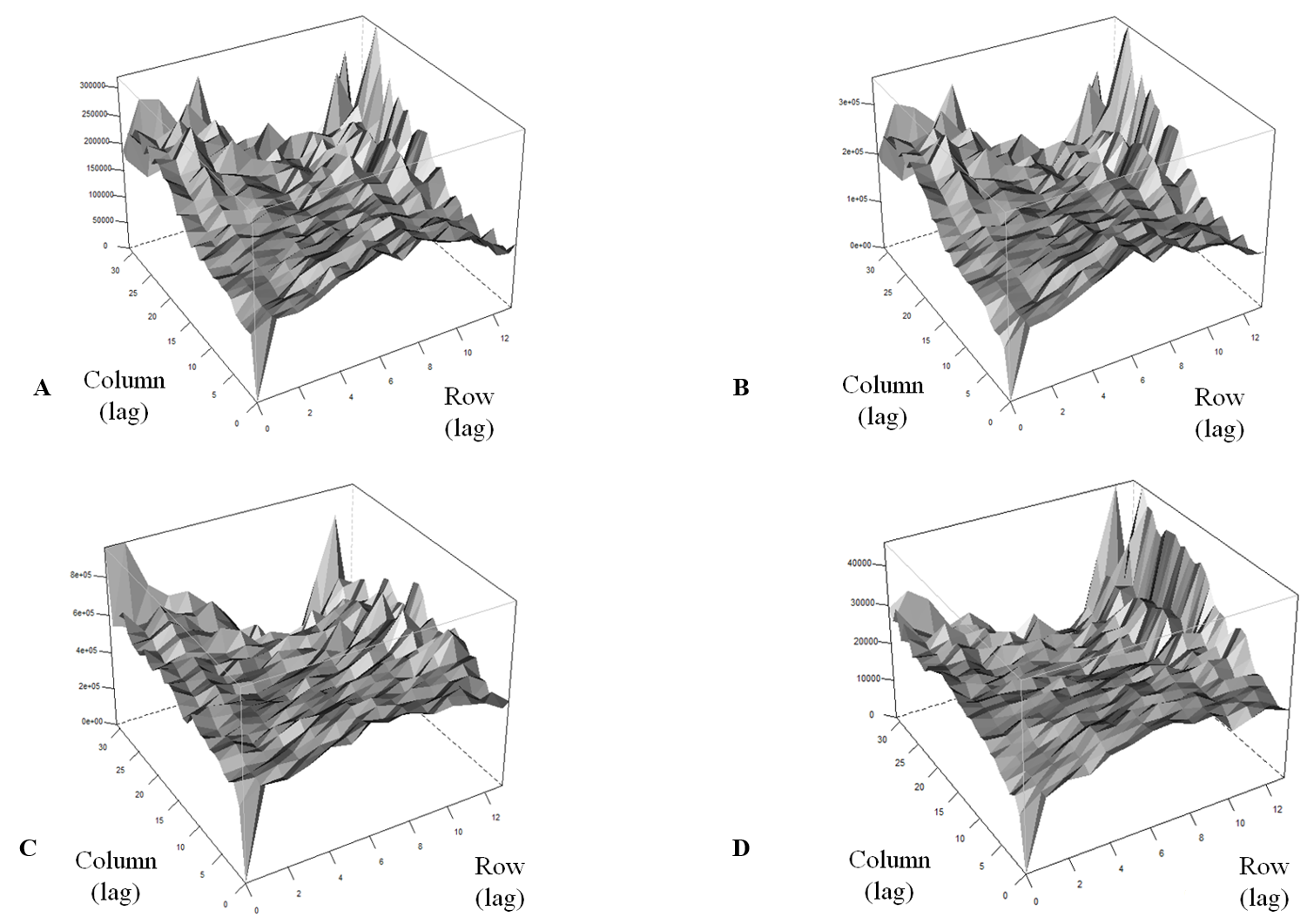


**Supplementary Figure 5.** Changes in variograms for herbage accumulation (HA) and normalized difference vegetation index (NDVI) before (A, C) and after (B, D) the inclusion of terms to control local and global trends for alfalfa yield in harvest two in an alfalfa breeding trial evaluated in Citra, FL. A) HA-base model, B) HA-spatial model, C) NDVI-base model, D) NDVI-spatial model. Row and Column are coordinates for the rows and columns in the experimental area, respectively.


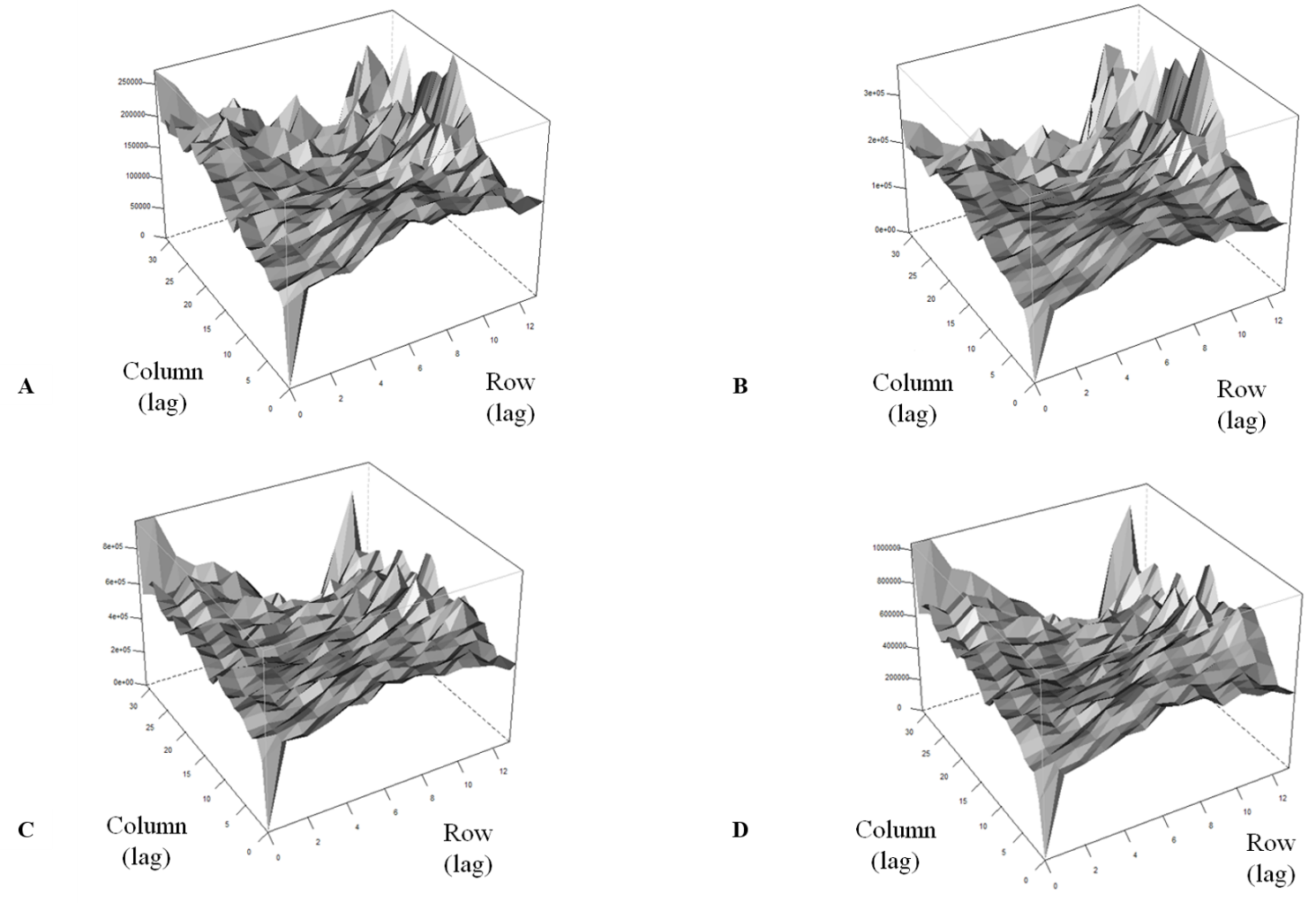


**Supplementary Figure 6.** Changes in variograms for herbage accumulation (HA) and normalized difference vegetation index (NDVI) before (A, C) and after (B, D) the inclusion of terms to control local and global trends for alfalfa yield in harvest three in an alfalfa breeding trial evaluated in Citra, FL. A) HA-base model, B) HA-spatial model, C) NDVI-base model, D) NDVI-spatial model. Row and Column are coordinates for the rows and columns in the experimental area, respectively.


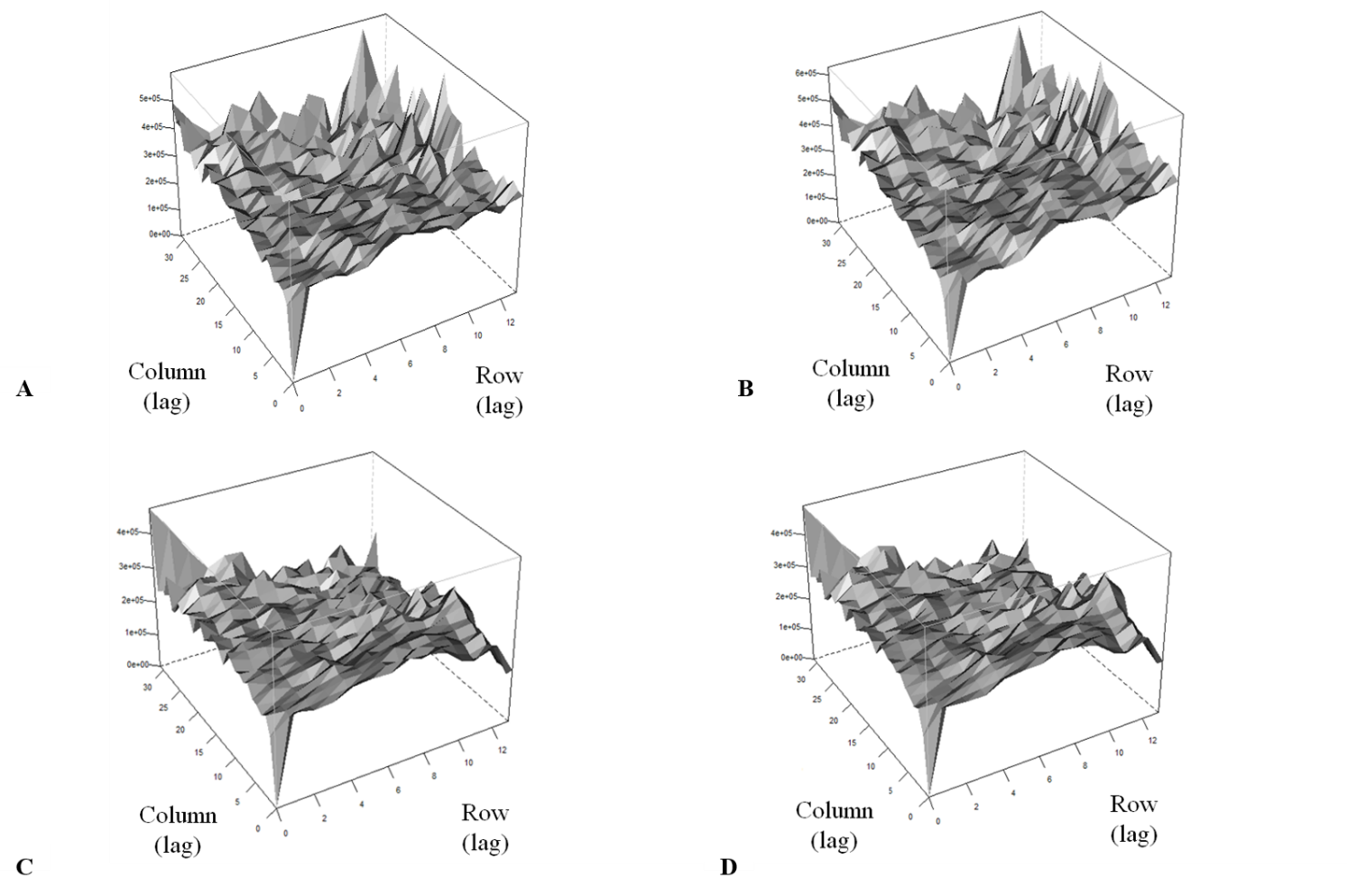


**Supplementary Figure 7.** Changes in variograms for herbage accumulation (HA) and normalized difference vegetation index (NDVI) before (A, C) and after (B, D) the inclusion of terms to control local and global trends for alfalfa yield in harvest four in an alfalfa breeding trial evaluated in Citra, FL. A) HA-base model, B) HA-spatial model, C) NDVI-base model, D) NDVI-spatial model. Row and Column are coordinates for the rows and columns in the experimental area, respectively.

## Supplementary Tables

**TABLE 1.** Estimates of the autocorrelation parameters for row ($\boldsymbol{\rho}_{\boldsymbol{r}}$) and columns ($\boldsymbol{\rho}_{\boldsymbol{C}}$**)**, for the traits herbage accumulation (HA), NDVI, and for the Bivariate model, in four different harvests (H1, H2, H3, and H4).

| **Harvest** | **autocorrelation** | **Variance** |
| --- | --- | --- |
| HA_H1 | $\boldsymbol{\rho}_{\boldsymbol{r}}$ | 0.10* |
| HA_H1 | $\boldsymbol{\rho}_{\boldsymbol{C}}$ | 0.33* |
| NDVI_H1 | $\boldsymbol{\rho}_{\boldsymbol{r}}$ | 0.10^ns^ |
| NDVI_H1 | $\boldsymbol{\rho}_{\boldsymbol{C}}$ | 0.35** |
| Bivariate_H1 | $\boldsymbol{\rho}_{\boldsymbol{r}}$ | 0.08^ns^ |
| Bivariate_H1 | $\boldsymbol{\rho}_{\boldsymbol{C}}$ | 0.42** |
| HA_ H2 | $\boldsymbol{\rho}_{\boldsymbol{r}}$ | 0.10^ns^ |
| HA_ H2 | $\boldsymbol{\rho}_{\boldsymbol{C}}$ | 0.36** |
| NDVI_H2 | $\boldsymbol{\rho}_{\boldsymbol{r}}$ | 0.10^ns^ |
| NDVI_ H2 | $\boldsymbol{\rho}_{\boldsymbol{C}}$ | 0.34** |
| Bivariate_H2 | $\boldsymbol{\rho}_{\boldsymbol{r}}$ | 0.13* |
| Bivariate_H2 | $\boldsymbol{\rho}_{\boldsymbol{C}}$ | 0.41* |
| HA_ H3 | $\boldsymbol{\rho}_{\boldsymbol{r}}$ | 0.10^ns^ |
| HA_ H3 | $\boldsymbol{\rho}_{\boldsymbol{C}}$ | 0.32*** |
| NDVI_ H3 | $\boldsymbol{\rho}_{\boldsymbol{r}}$ | 0.10^ns^ |
| NDVI_ H3 | $\boldsymbol{\rho}_{\boldsymbol{C}}$ | 0.39*** |
| Bivariate_H3 | $\boldsymbol{\rho}_{\boldsymbol{r}}$ | 0.10^ns^ |
| Bivariate_H3 | $\boldsymbol{\rho}_{\boldsymbol{C}}$ | 0.46** |
| HA_ H4 | $\boldsymbol{\rho}_{\boldsymbol{r}}$ | 0.10^ns^ |
| HA_ H4 | $\boldsymbol{\rho}_{\boldsymbol{C}}$ | 0.33* |
| NDVI_ H4 | $\boldsymbol{\rho}_{\boldsymbol{r}}$ | 0.01^ns^ |
| NDVI_ H4 | $\boldsymbol{\rho}_{\boldsymbol{C}}$ | 0.34** |
| Bivariate_H4 | $\boldsymbol{\rho}_{\boldsymbol{r}}$ | 0.05^ns^ |
| Bivariate_H4 | $\boldsymbol{\rho}_{\boldsymbol{C}}$ | 0.28** |

*significance at *p*<0.05, ** significance at p<0.01, and *** significance at p<0.001, ns = not significant

**TABLE 2.** Akaike information criteria (AIC) and Bayesian Information Criteria (BIC) from base and spatial models among four harvests

| Harvest | Model | AIC | BIC |
| --- | --- | --- | --- |
| 1 | HA Base | 5963 | 5979.3 |
| 1 | HA Spatial | 5909 | 5928.8 |
| 1 | HTP Base | 6906.1 | 6922.2 |
| 1 | HTP Spatial | 6810.2 | 6830.4 |
| 2 | HA Base | 5617 | 5633.1 |
| 2 | HA Spatial | 5573 | 5597.3 |
| 2 | HTP Base | 4942 | 4958.2 |
| 2 | HTP Spatial | 4896 | 4920.2 |
| 3 | HA Base | 5793 | 5809.2 |
| 3 | HA Spatial | 5730 | 5750.4 |
| 3 | HTP Base | 6337 | 6353.1 |
| 3 | HTP Spatial | 6268 | 6292.3 |
| 4 | HA Base | 6009 | 6025.3 |
| 4 | HA Spatial | 5985 | 6009.5 |
| 4 | HTP Base | 6040 | 6056.6 |
| 4 | HTP Spatial | 6012 | 6036.7 |

**TABLE 3.** Genetic gain based on BLUP with standard deviation before converting it into percentage from four harvests

| **Model** | **Missing_HA** | **Genetic Gain (BLUPS)** | **SD** | **Harvest** |
| --- | --- | --- | --- | --- |
| bivariate | 10 | 1966.8 | 18.6 | 1 |
| bivariate | 20 | 1977 | 38.6 | 1 |
| bivariate | 30 | 1972.6 | 40 | 1 |
| bivariate | 40 | 1956.6 | 47.4 | 1 |
| bivariate | 50 | 1971.1 | 51 | 1 |
| bivariate | 60 | 1950.3 | 56.9 | 1 |
| bivariate | 70 | 1966.1 | 82.4 | 1 |
| univariate_DMY | 10 | 1861.9 | 44.2 | 1 |
| univariate_DMY | 20 | 1851.6 | 61.9 | 1 |
| univariate_DMY | 30 | 1813.9 | 67.2 | 1 |
| univariate_DMY | 40 | 1793.8 | 109.2 | 1 |
| univariate_DMY | 50 | 1806.2 | 148.4 | 1 |
| univariate_DMY | 60 | 1713.2 | 151.3 | 1 |
| univariate_DMY | 70 | 1709.2 | 206 | 1 |
| univariate_NDVI | 10 | 1789.8 | 31.41 | 1 |
| univariate_NDVI | 20 | 1829 | 35.78 | 1 |
| univariate_NDVI | 30 | 1770.5 | 57.42 | 1 |
| univariate_NDVI | 40 | 1789.7 | 79.42 | 1 |
| univariate_NDVI | 50 | 1729.5 | 94.59 | 1 |
| univariate_NDVI | 60 | 1719.5 | 130.6 | 1 |
| univariate_NDVI | 70 | 1638.2 | 148.26 | 1 |
| bivariate | 10 | 970.48 | 14.15 | 2 |
| bivariate | 20 | 978.77 | 24.4 | 2 |
| bivariate | 30 | 976.49 | 33.49 | 2 |
| bivariate | 40 | 973.39 | 41.3 | 2 |
| bivariate | 50 | 971.86 | 52.52 | 2 |
| bivariate | 60 | 961.94 | 56.55 | 2 |
| bivariate | 70 | 990.33 | 80.93 | 2 |
| univariate_DMY | 10 | 953.46 | 37.7 | 2 |
| univariate_DMY | 20 | 947.16 | 57.82 | 2 |
| univariate_DMY | 30 | 937.44 | 74.67 | 2 |
| univariate_DMY | 40 | 938.6 | 83.84 | 2 |
| univariate_DMY | 50 | 911.28 | 83.7 | 2 |
| univariate_DMY | 60 | 878.5 | 101.57 | 2 |
| univariate_DMY | 70 | 855.82 | 140.74 | 2 |
| univariate_NDVI | 10 | 854.14 | 25.14 | 2 |
| univariate_NDVI | 20 | 849.92 | 40.34 | 2 |
| univariate_NDVI | 30 | 826.71 | 56.06 | 2 |
| univariate_NDVI | 40 | 822.25 | 61.18 | 2 |
| univariate_NDVI | 50 | 794.87 | 57.09 | 2 |
| univariate_NDVI | 60 | 824.68 | 85.28 | 2 |
| univariate_NDVI | 70 | 816.63 | 90.28 | 2 |
| bivariate | 10 | 1306.07 | 16.82 | 3 |
| bivariate | 20 | 1307.21 | 26.79 | 3 |
| bivariate | 30 | 1307.16 | 32.29 | 3 |
| bivariate | 40 | 1303.7 | 39.02 | 3 |
| bivariate | 50 | 1283.5 | 38.42 | 3 |
| bivariate | 60 | 1307.19 | 56.21 | 3 |
| bivariate | 70 | 1283.29 | 68.7 | 3 |
| univariate_DMY | 10 | 1302.34 | 39.37 | 3 |
| univariate_DMY | 20 | 1298.37 | 54.98 | 3 |
| univariate_DMY | 30 | 1272.91 | 78.97 | 3 |
| univariate_DMY | 40 | 1234.67 | 92.19 | 3 |
| univariate_DMY | 50 | 1216.5 | 90.26 | 3 |
| univariate_DMY | 60 | 1213.99 | 106.66 | 3 |
| univariate_DMY | 70 | 1247.71 | 169.07 | 3 |
| univariate_NDVI | 10 | 1186.11 | 23.86 | 3 |
| univariate_NDVI | 20 | 1179.22 | 41.36 | 3 |
| univariate_NDVI | 30 | 1168.11 | 47.28 | 3 |
| univariate_NDVI | 40 | 1149.54 | 73.46 | 3 |
| univariate_NDVI | 50 | 1165.95 | 74.98 | 3 |
| univariate_NDVI | 60 | 1114.9 | 79.03 | 3 |
| univariate_NDVI | 70 | 1140.34 | 103.72 | 3 |
| bivariate | 10 | 1791.92 | 31.8 | 4 |
| bivariate | 20 | 1798.39 | 57.73 | 4 |
| bivariate | 30 | 1766.33 | 63.19 | 4 |
| bivariate | 40 | 1753.19 | 90.65 | 4 |
| bivariate | 50 | 1757.45 | 88.76 | 4 |
| bivariate | 60 | 1730.35 | 109.16 | 4 |
| bivariate | 70 | 1762.37 | 130.51 | 4 |
| univariate_DMY | 10 | 1764.99 | 43.17 | 4 |
| univariate_DMY | 20 | 1741.17 | 64.2 | 4 |
| univariate_DMY | 30 | 1718.95 | 107.61 | 4 |
| univariate_DMY | 40 | 1715.76 | 118.78 | 4 |
| univariate_DMY | 50 | 1645.99 | 142.5 | 4 |
| univariate_DMY | 60 | 1648.91 | 198.27 | 4 |
| univariate_DMY | 70 | 1649.17 | 214.11 | 4 |
| univariate_NDVI | 10 | 1571.53 | 35.4 | 4 |
| univariate_NDVI | 20 | 1552.48 | 56.55 | 4 |
| univariate_NDVI | 30 | 1557.68 | 55.15 | 4 |
| univariate_NDVI | 40 | 1549.35 | 98.09 | 4 |
| univariate_NDVI | 50 | 1527.11 | 77.56 | 4 |
| univariate_NDVI | 60 | 1503.57 | 98.84 | 4 |
| univariate_NDVI | 70 | 1491.02 | 126.29 | 4 |
